# Study on the reduction of residual stress in laser cladding layers through groove texture

**DOI:** 10.1038/s41598-024-66793-5

**Published:** 2024-07-10

**Authors:** Changlong Zhao, Chen Ma, Weilong Du, Zice Yu, Zihao Zhang

**Affiliations:** College of Mechanical and Vehicle Engineering, Changchun University, Changchun, 130022 China

**Keywords:** Laser cladding, Surface texture, Residual stress, Surface roughness, Mechanical engineering, Engineering

## Abstract

In order to develop a method for the production of crack-free cladding layers, we combined surface texturing technology with laser cladding, establishing a multi-field coupled numerical simulation model. A separate investigation was conducted into the temperature, stress, and fluid fields in laser cladding processes with and without texturing, seeking optimal cladding parameters, and conducted experiments. The results of the numerical simulations indicate that pre-set texturing effectively reduces the temperature gradient during the cladding process, thereby making the thermal cycle curve smoother. The residual stresses in the X, Y, and Z directions are reduced by 34.84%, 3.94%, and 50.22%, respectively. The introduction of texturing reduces the internal flow velocity of the melt pool, preventing the occurrence of a double vortex effect. Experimental results show that the residual stresses in the X, Y, and Z directions of the predefined textured cladding layer are reduced by approximately 41%, 8%, and 47%, respectively, compared to the non-textured cladding layer. This effectively improves the surface roughness and internal grain size of the cladding layer, with no significant defects at the metallurgical bonding positions, providing a reference for future improvements in cladding layer quality.

## Introduction

45 steel is a high-quality medium carbon quenched and tempered structural steel with low levels of harmful impurities such as sulfur and phosphorus. It exhibits strong comprehensive mechanical properties and excellent cold and hot processing performance. It is frequently employed in the manufacture of critical components under high-intensity dynamic conditions, including pistons, shafts, gears, connecting rods, and bolts in compressors. These key components are prone to surface wear, corrosion, oxidation, and fracture during operation, which can result in failure. Therefore, the repair of failed parts is imperative^1–3^

Laser cladding technology is an innovative surface improvement technique based on the "discrete + stacking" concept. It breaks away from traditional material removal processes. However, the rapid heating and cooling characteristics of the process result in a significant temperature gradient between the cladding layer and the substrate. This results in the formation of high residual stresses within the cladding layer, causing defects such as cracks and pores. These limitations hinder the further development of laser cladding technology^4–6^.

Currently, researchers worldwide are optimizing the surface quality of cladding layers by controlling processing parameters and introducing auxiliary processing techniques^7–9^. Ren et al.^10^ designed an orthogonal experimental plan, determining those factors affecting quality of the cladding layer, in descending order, are powder feeding speed, scanning speed, and laser power. The optimal cladding parameters were found to be laser power of 1400 W, scanning speed of 7 mm/s, and powder feeding speed of 21 g/min. Under these optimal parameters, the cladding layer exhibited a microhardness of 1252HV, a wear rate of 1.5 × 10^−5^ mm^3^/(N m), and significantly improved wear resistance. Shi et al.^11^ applied laser cladding of Ni60a/SiC coating on 65Mn blades, with the optimal cladding parameters being laser power 2000 W, scanning speed 400 mm/min, and spot diameter 3 mm. The resulting cladding layer exhibited a microhardness of 870HV0.1. Compared to the substrate, the friction coefficient was found to have decreased by 29%, the scratch depth decreased by 83%, and the wear resistance significantly increased. Todaro et al.^12,13^ introduced ultrasound during the laser cladding process to manipulate the grain structure of 316L stainless steel and Ti-6Al-4V, successfully achieving a complete transformation from coarse columnar grains to equiaxed grains. Ning et al.^14^ investigated the influence of different ultrasonic frequencies on the microstructure of laser-cladded layers, their findings indicated that variations in ultrasonic frequency affect the size of the molten pool, temperature distribution, and peak temperature. Lei et al.^15^ validated the stirring effect of an electromagnetic composite field on the molten pool, achieving a transformation and refinement of the microstructure in the Fe901 cladding layer. Xu^16^ introduced a combined electromagnetic and ultrasonic energy field into the laser molten pool, discovering a mutual enhancement effect that resulted in the effective homogenization and refinement of the cladding layer's composition and microstructure. Han et al.^17,18^ applied high-frequency ultrasonic forging on the soon-to-solidify cladding layer, inducing recrystallization in the microstructure for grain refinement and subsequently enhancing surface performance.

With the accessibility of microfabrication techniques in recent years, surface texture technology has effectively promoted the flourishing development of tribology^19–21^. Many scholars have analysed the application effects and development potential of surface texture in various aspects including the reduction of friction and wear, lubricating, the reduction of resistance, and the extension of anti-corrosion life on friction surfaces^22,23^. PEI et al.^24^ employed finite element methods to study the impact of nine surface textures on the lubrication performance of floating ring bearings. The results revealed that surface texture significantly influences the lubrication performance of bearings, with concave textures being more suitable for engineering applications. Huang et al.^25^, by prepared micro-groove textures on the fixed rotor surface of an oil extraction hydraulic motor, which effectively improved lubrication conditions, reduced the friction coefficient of the friction pair, and facilitated the smooth start-up of the hydraulic-driven screw pump system. Many researchers have also pre-set surface textures on cutting tools to enhance their lifespan. Xiang et al.^26^ pre-set surface textures on hard alloy cutting tools, and the results indicated that these tools could effectively enhance the bonding strength of the film and improve the wear resistance of the tools. KAWASEGI N et al.^27^ employed FIB methods to process concave textures on diamond cutting tools and, when cutting Al-A5052 workpieces, the research results demonstrated that textured tools could enhance the surface quality of the machined workpieces. However, there are few reports on applying surface texture to laser cladding technology.

In this study, 45 steel was chosen as the substrate, and 316L stainless steel powder was selected as the cladding powder. Surface texture technology was integrated with laser cladding technology, and an experimental plan was designed to investigate the influence of surface texture technology on the residual stress of the cladding layer. This provides a theoretical foundation for subsequent laser cladding experiments.

## Numerical simulation

### Material selection

**Figure 1:**
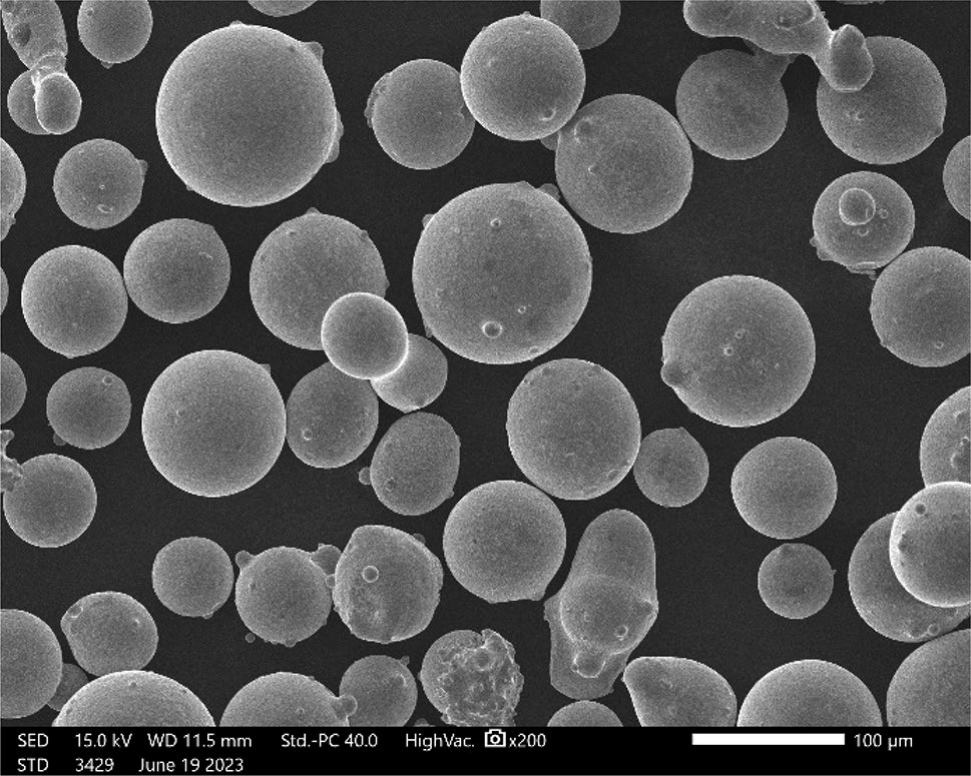
Morphological details of 316L powder.

**Figure 2:**
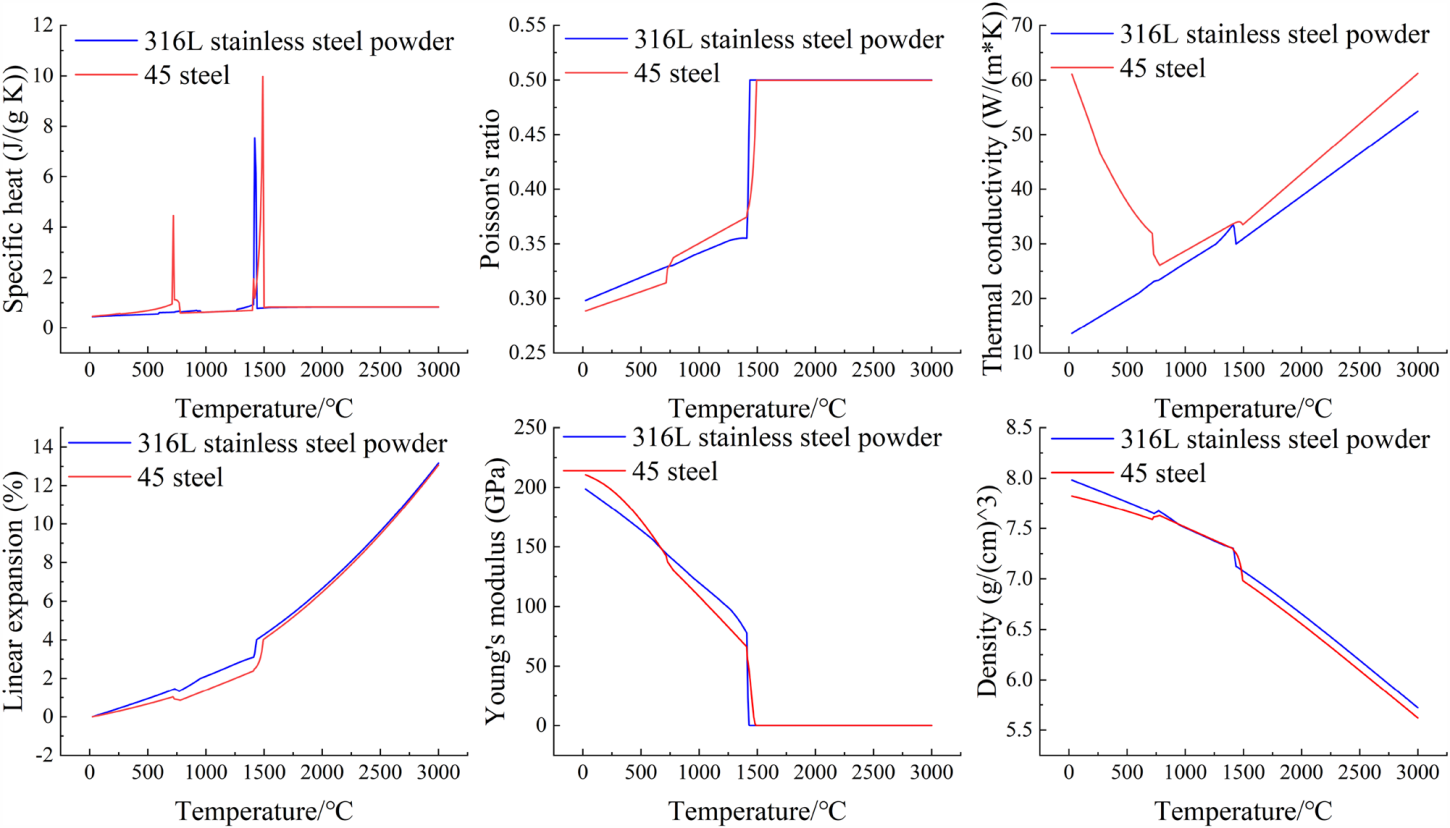
Thermal-physical properties of 316L stainless steel.

The numerical simulation employs 45 steel as the matrix, with its elemental composition presented in Table 1. The powder used is 316L stainless steel, and its elemental composition is detailed in Table 2. The morphology of the powder is illustrated in Fig. 1. The thermal-physical properties of 45 steel and 316L stainless steel powder with temperature variations were calculated using the incremental integration method in JMatPro software. The results were imported into Origin software for visualization, as depicted in Fig. 2.

**Table 1:** Elemental composition of 45 steel.

Element	C	Cr	Mn	Ni	P	S	Si	Fe
Mass fraction (%)	0.42	0.25	0.50	0.25	0.035	0.035	0.17	98.34

**Table 2:** Elemental composition of 316L stainless steel.

Element	Fe	Cr	Mn	Mo	Ni	Si	C
Mass fraction (%)	64.447	17.3	1.74	2.66	13.1	0.73	0.023

### Multi-field coupling numerical simulation technique

The multi-field coupling numerical simulation technique is an advanced method of coupling different physical fields for the purpose of conducting simulation analysis^28^. Through multi-field coupling numerical simulation, it is possible to more accurately simulate real-world scenarios, including the interactions between different physical fields, thereby improving the accuracy and reliability of the simulation results. In this study, temperature field, stress field, and fluid field are coupled to numerically simulate the laser cladding process. This comprehensive analysis enables a better understanding and evaluation of the laser cladding process, revealing the mechanisms by which surface texture technology influences the performance of the cladding layer.

### Overall control equations for laser cladding process

The overall control equations for the laser cladding process^29^ are given by

1 $$\frac{\partial \rho }{{\partial t}} + \nabla \cdot \left( {\rho v} \right) = 0$$

2 $$\rho \left[ {\frac{\partial v}{{\partial t}} + \left( {v \cdot \nabla } \right)v} \right] = \nabla \cdot \left[ { - pI + \mu \left( {\nabla v + \left( {\nabla v} \right)^{T} } \right) - \frac{2\mu }{3}\left( {\nabla \cdot v} \right)I} \right] - K_{0} \frac{{\left( {1 - f_{l} } \right)^{2} }}{{f_{l}^{3} + B}}v$$

3 $$\rho C_{p} \frac{\partial T}{{\partial t}} + \rho C_{p} v \cdot \nabla T = \nabla \cdot \left( {k\nabla T} \right) - \frac{\partial H}{{\partial t}} - \rho v \cdot \nabla H$$

4 $$\rho \frac{{\partial \xi^{2} }}{{\partial t^{2} }} = \nabla \cdot S + FV$$

In the equations, $\rho$ represents density, $t$ represents time, $v$ represents the metal flow velocity within the melt pool, $\mu$ represents the fluid dynamic viscosity, $p$ represents pressure, $T$ represents temperature, $C_{p}$ represents specific heat capacity, $k$ represents thermal conductivity, $\xi$ is the displacement vector, $F$ represents constraint forces, and $S$ represents the total displacement. Equation (1) is the continuity equation, and Eq. (2) is the Navier–Stokes momentum equation, where $K_{0}$ is a constant determined by the porous morphology and $B$ is a minimal parameter that avoids the denominator to be zero. Equation (3) is the energy equation, and $H$ represents the latent heat of metal fusion, which is denoted as $\Delta H = Lf_{l}$. The liquid mass fraction is represented as $f_{l}$.

5 $$f_{l} = \left\{ \begin{gathered} 1,T > T_{l} \hfill \\ \frac{{T - T_{S} }}{{T_{L} - T_{S} }},T_{S} \le T \le T_{l} \hfill \\ 0,T < T_{S} \hfill \\ \end{gathered} \right.$$

In the equations, the subscripts $S$ and $l$ represent the solid phase and liquid phase, respectively. Equation (4) represents the stress equation.

In the laser cladding process, the thermal stress variations induced by high temperatures are highly complex. This study employs the principle of incremental elastic deformation for analysis and resolution. The strain at any point on the workpiece can be expressed using Eq. (6):

6 $$d\varepsilon = d\varepsilon_{c} + d\varepsilon_{p} + d\varepsilon_{th}$$

In the equation, $d\varepsilon$ represents the total strain, $d\varepsilon_{c}$ represents the elastic strain, $d\varepsilon_{p}$ represents the plastic strain, and $d\varepsilon_{th}$ represents the thermal strain.

The thermal strain of the workpiece is calculated using the following formula:

7 $$d\varepsilon_{th} = \alpha \left( {T - T_{ref} } \right)$$

In the equation, $\alpha$ denotes the material's coefficient of linear expansion, which varies with temperature. When the temperature variation is small, $\alpha$ can be considered constant. $T_{ref}$ is the reference temperature.

The plastic thermal stress of the workpiece is calculated using the following formula:

8 $$d\sigma = \left[ D \right]_{cp} \left( {d\varepsilon_{th} - d\varepsilon_{0} } \right) - d\sigma_{0}$$

In the equation, $d\sigma$ represents the plastic thermal stress, $\left[ D \right]_{cp}$ represents the thermoplastic matrix, $d\varepsilon_{0}$ represents the initial strain, and $d\sigma_{0}$ represents the initial stress.

### Heat source selection

For the numerical simulation of laser cladding in this study, the heat source is selected using the Moving Heat plugin in ANSYS Workbench. The heat source equation defined by this plugin is as follows

9 $$q = C_{2} e^{{ - \frac{{\left[ {\left( {x - x_{0} } \right)^{2} + \left( {y - y_{0} } \right)^{2} + \left( {z - z_{0} } \right)^{2} } \right]}}{{C_{1}^{2} }}}}$$

In the equation, $q$ represents the heat flux, $C_{1}$ represents the beam radius, $C_{2}$ represents the laser power, and $\left( {x_{0} ,y_{0} ,z_{0} } \right)$ represents the instantaneous position of the heat source center.

**Figure 3:**
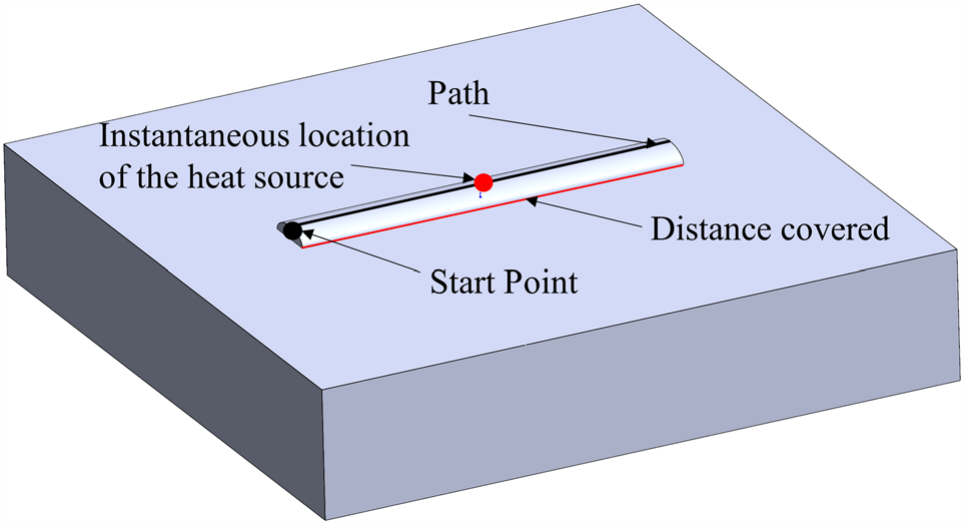
Schematic diagram of heat source application.

The heat source application principle of the Moving Heat plugin is shown in Fig. 3.

### Finite element model establishment

Modeling assumptions for the laser cladding process are as follows:

1. The metal flow in the molten pool is assumed to be laminar and incompressible Newtonian fluid.
2. The energy distribution of the laser beam within the spot follows a Gaussian distribution, and the power is constant.
3. The material is considered isotropic.
4. The concentration of powder flow is assumed to follow a Gaussian distribution, and the powder that is dropped into the molten pool immediately melts.
5. The clad material is assumed to follow the Von Mises yield criterion.

Substrate dimensions are 50 mm × 50 mm × 10 mm, and coating dimensions are 4 mm × 30 mm × 1 mm. Due to the influence of laser cladding system parameters, the best results are achieved with a spot radius of 2 mm. Based on this constraint, grooved texture has been designed, taking into account both the depth and width of the texture's impact on the cladding layer's performance. When the depth is too shallow, there is minimal change in the surface morphology of the clad layer, and the texture fails to have the desired effect. When the depth is excessive, the cladding layer is below the substrate plane, negating the additive manufacturing effect. According to the preliminary simulation and experimental results of the research team, the grooved texture performs best with dimensions of 2 mm × 50 mm × 0.5 mm.

**Figure 4:**
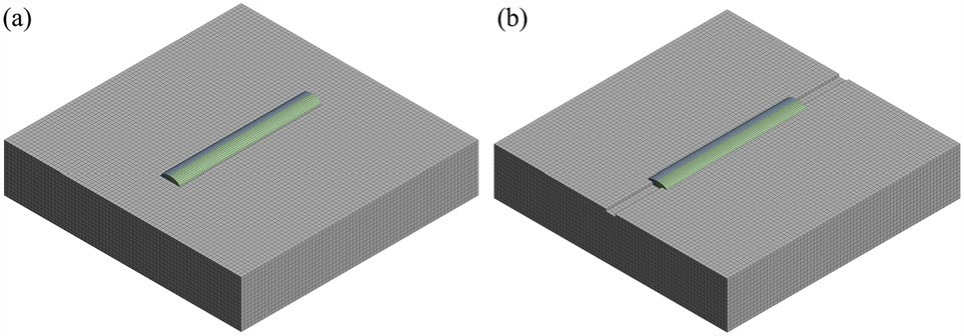
(**a**) Meshing pattern without surface texture; (**b**) meshing pattern with pre-designed surface texture.

The model was constructed using SolidWorks 3D software, and the mesh was generated for the model. To enhance the computational accuracy and speed, the mesh was refined, with the clad layer mesh being denser and the substrate mesh being sparser. This not only enhances computational speed but also ensures accuracy. The mesh division is illustrated in Fig. 4. The contact surface below the substrate adopts a nonlinear convective heat transfer coefficient for metallic contact, while the coefficients for other surfaces are set to the nonlinear convective heat transfer coefficient for air.

### Numerical simulation experimental program design

Based on preliminary experimental preparations, laser powers were varied at 600 W, 800 W, 1000 W, 1200 W, and 1400 W. Scan speeds were set at 1 mm/s, 2 mm/s, 3 mm/s, 4 mm/s, and 5 mm/s, with a spot radius of 2 mm. The experimental plan is detailed in Table 3.

**Table 3:** Numerical simulation experimental scheme.

Parameter	Level 1	Level 2	Level 3	Level 4	Level 5
Laser power (W)	600	800	1000	1200	1400
Scanning speed (mm/s)	1	2	3	4	5
Spot radius (mm)	2	2	2	2	2

## Analysis of results

### Analysis of temperature field results

**Figure 5:**
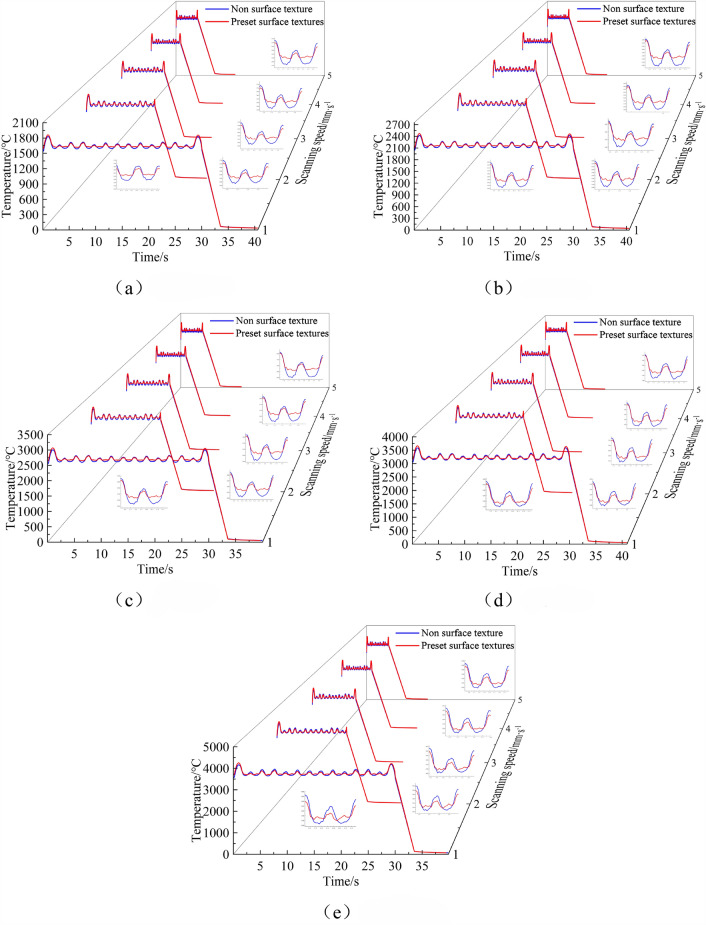
Simulation results of temperature field under different melting parameters. (**a**) 600W. (**b**) 800W. (**c**) 1000W. (**d**) 1200W. (**e**) 1400W.

Laser cladding is a rapid prototyping technology, where the core involves using a high-power laser beam to melt powder material and deposit it on the substrate surface, forming parts of the desired shape. To achieve a high-quality clad layer, precise control of the temperature field during the laser cladding process is essential. Using ANSYS Workbench software, temperature field simulations were conducted for laser cladding with and without texture under different parameters, and the simulation results are presented in Fig. 5.

Figure 5 shows the thermal cycle curves under different cladding parameters. Observing Fig. 5, it can be noted that when the scanning speed is constant, the peak temperature in the thermal cycle curve increases with the continuous increase of the laser power, and the peak temperature is positively correlated with laser power. When the laser power is constant, with the continuous increase of scanning speed, the peak temperature in the thermal cycle curve decreases, and the peak temperature is negatively correlated with the scanning speed.

Under the same overlay conditions, comparing the temperature fields during the overlay process with and without pre-set textures, it is found that pre-set textures can effectively reduce the maximum temperature during the overlay process, increase the minimum temperature, and make the temperature during the overlay process more stable. As the laser power and scanning speed remain constant, and the energy output of the laser per unit time is fixed, pre-set textures effectively increase the contact area between the laser and the substrate. Consequently, more laser energy is absorbed within the same time frame, resulting in a more stable temperature in the thermal cycle curve and reducing the temperature gradient during the overlay process.

### Analysis of temperature field results

**Figure 6:**
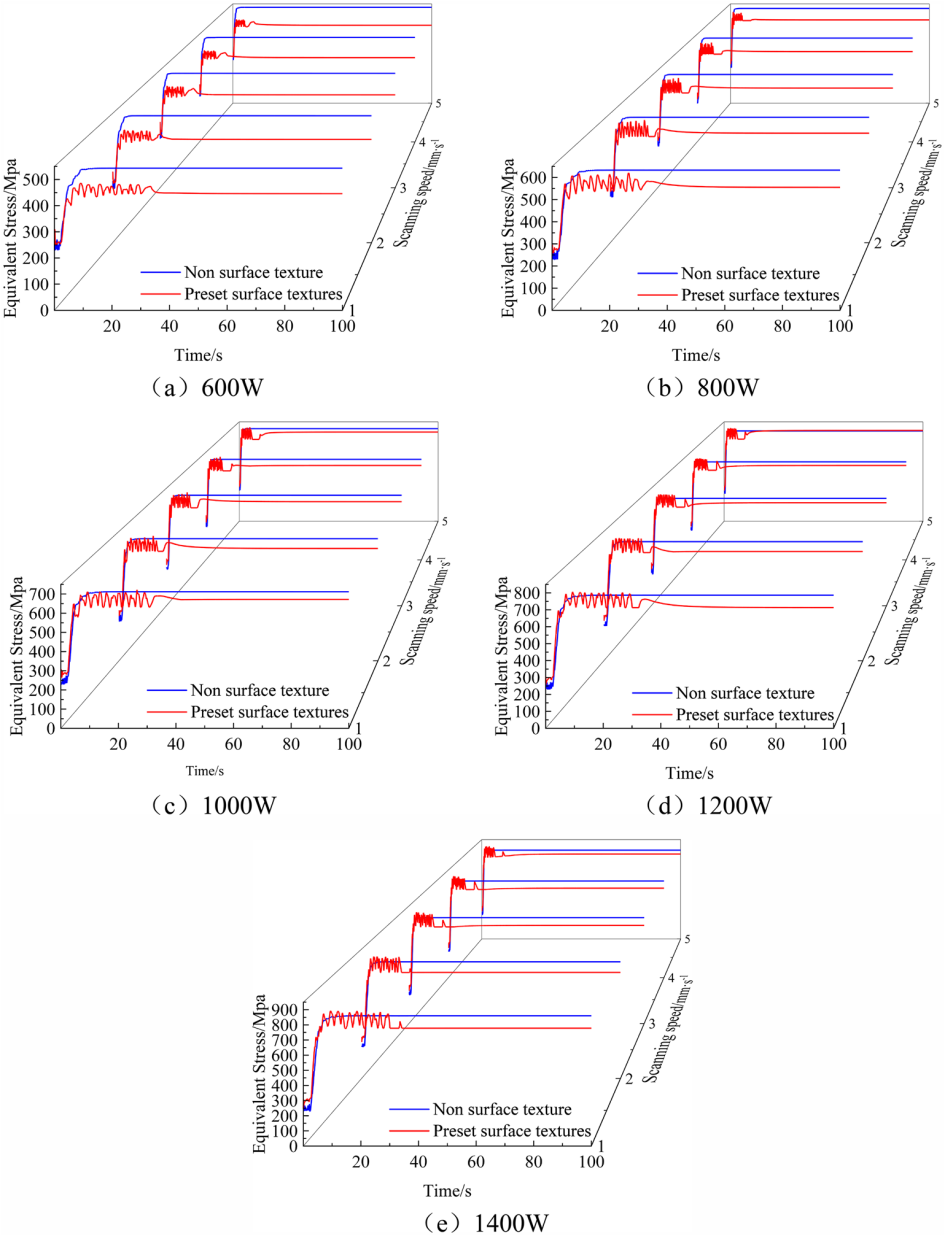
Simulation results of stress field under different cladding parameters. (**a**) 600W. (**b**) 800W. (**c**) 1000W. (**d**) 1200W. (**e**) 1400W.

Laser cladding is an emerging metal manufacturing process that can produce high-strength, high-performance metal components, but it also generates significant residual stress. Through ANSYS Workbench laser cladding stress field simulation, the internal stress distribution of the cladding layer can be predicted. Therefore, numerical simulations of the stress field with and without texture were conducted using ANSYS Workbench software. The simulation results of the laser cladding stress field for different experimental groups are extracted, as shown in Fig. 6.

**Figure 7:**
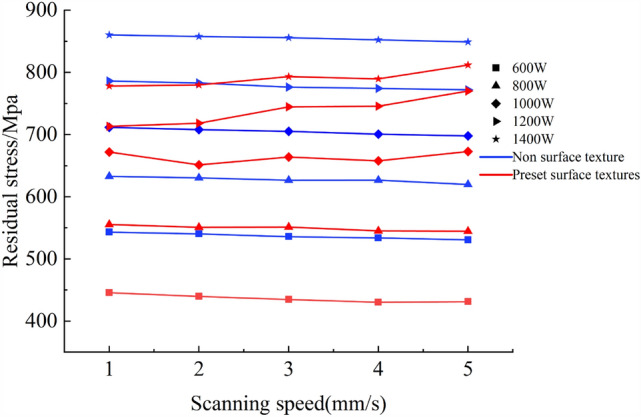
Comparison of residual stresses in each group.

**Figure 8:**
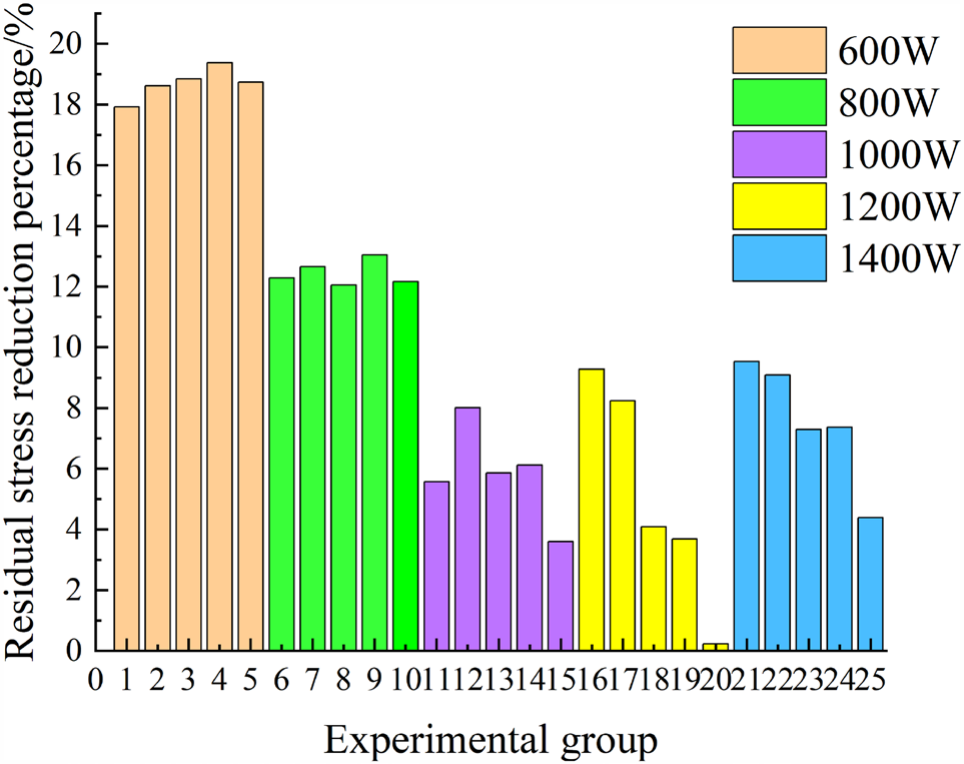
Percentage reduction of residual stress.

Figure 6 shows the residual stress curves under different cladding parameters. From Fig. 6, it can be observed that both textured and non-textured residual stress curves exhibit unstable alternating thermal stresses. The non-textured unstable alternating thermal stress is mainly present at the beginning of the cladding process, while the textured unstable alternating thermal stress occurs throughout the entire cladding process. In conjunction with this study, the presence of surface texture leads to the generation of unstable alternating thermal stress during the cladding process, significantly reducing the residual stress in the cladding layer, as seen in experimental groups 1, 2, 3, 4, etc., with very few cases approaching the numerical values of non-textured residual stress, such as experimental groups 15, 20, etc. For a more intuitive demonstration of the extent of residual stress reduction, the residual stress of each experimental group was extracted, and the percentage reduction in residual stress was calculated. The resulting plots are shown in Figs. 7 and 8.

**Figure 9:**
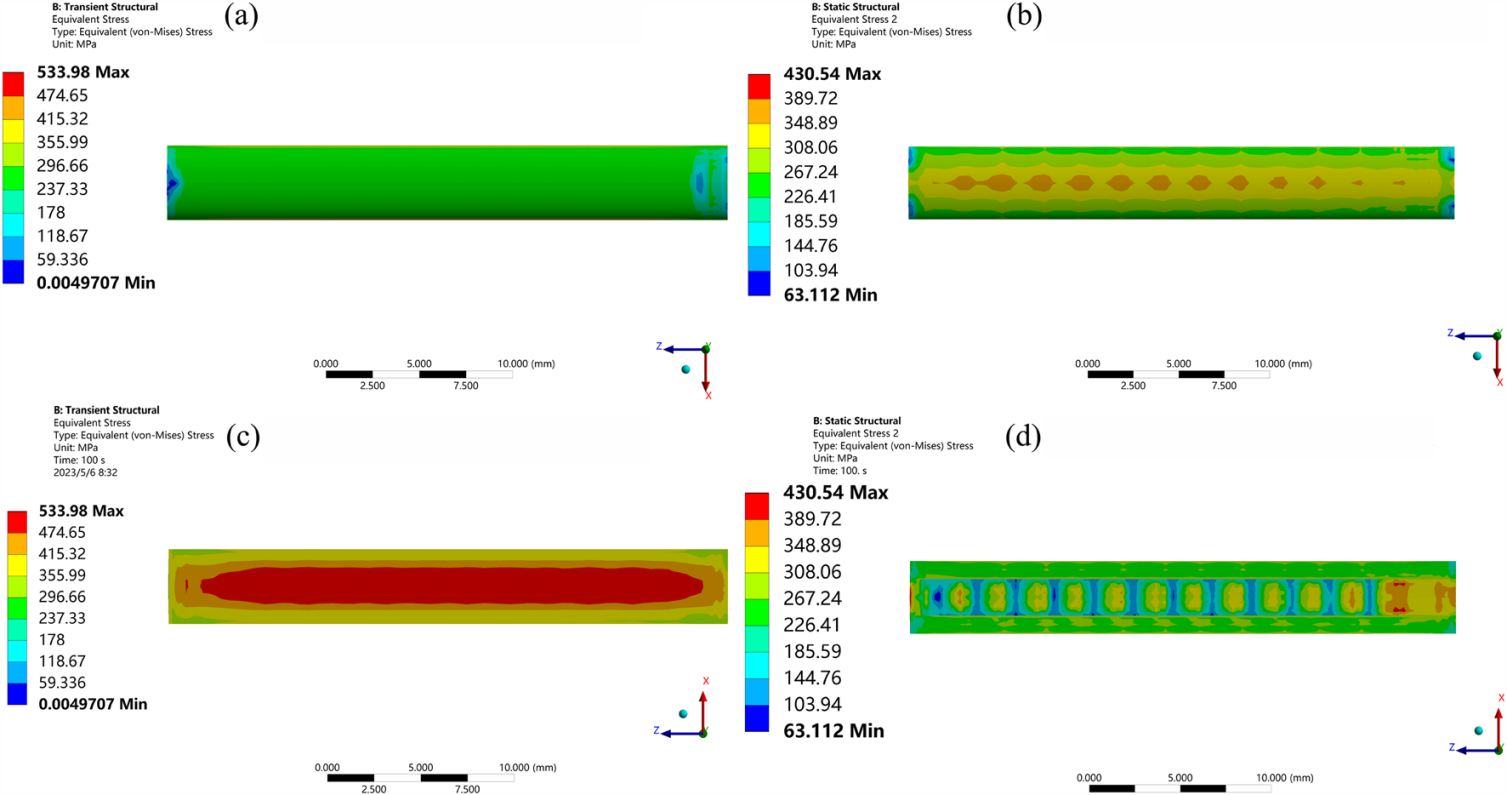
Residual stress contour maps of cladding layer with and without surface texture. (**a**) Non texture cladding surface. (**b**) Preset texture cladding surface. (**c**) Non texture cladding bottom. (**d**) Preset texture cladding bottom.

In Fig. 7, blue represents the non-textured experimental group, red represents the textured experimental group, and different powers are marked with different symbols. From Fig. 7, it can be observed that in the non-textured experimental group, with constant laser power, the residual stress of the cladding layer gradually decreases as the scanning speed increases. When the scanning speed is constant, the residual stress of the cladding layer gradually increases with the increase in laser power. In other words, the residual stress of the cladding layer is positively correlated with laser power and negatively correlated with the scanning speed. In the textured experimental group, when the laser power is 600 W and 800 W, the residual stress of the cladding layer gradually decreases with the increase of the scanning speed. When the laser power is 1000 W, the residual stress fluctuates but shows an overall upward trend. When the laser power is 1200 W and 1400 W, the residual stress of the cladding layer shows an upward trend. When the scanning speed is constant, with the increase in laser power, the residual stress of the cladding layer gradually increases. In other words, the residual stress of the cladding layer is positively correlated with the laser power, consistent with the trend in the non-textured experiment. From Fig. 8, it can be seen that the residual stress with pre-set texture decreases by a maximum of 19.37% compared to that without texture. The optimal cladding experimental group is No. 4, with cladding parameters of laser power 600 W, scanning speed 4 mm/s, and spot radius 2 mm. Extract the cloud map of residual stress distribution with and without texture for this group, as shown in Fig. 9.

**Figure 10:**
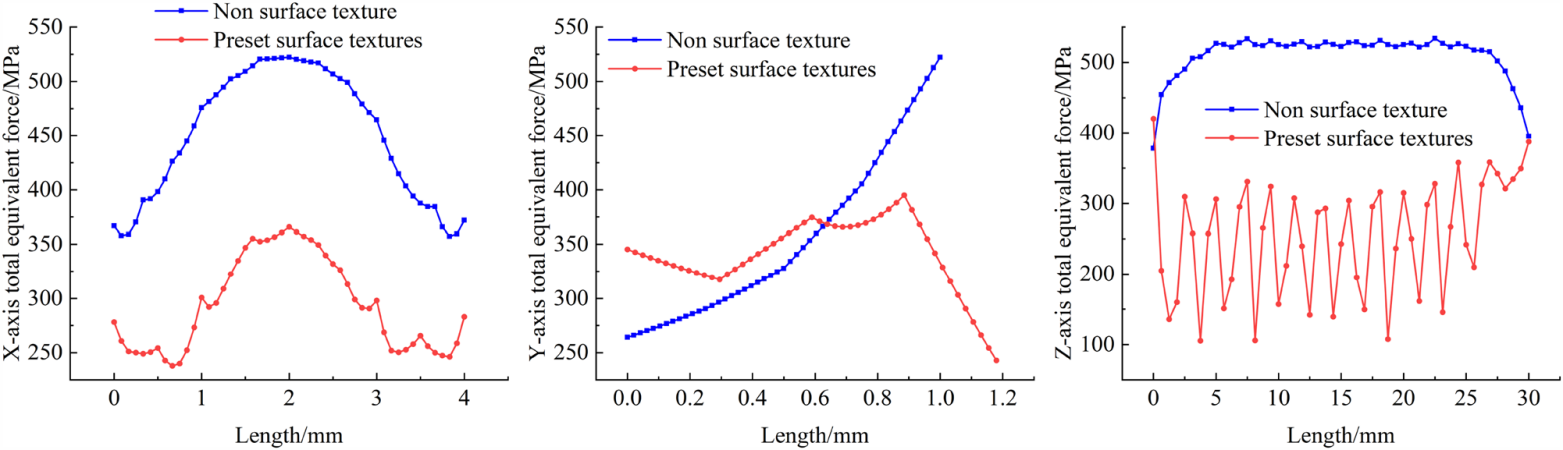
Residual stress curves in X, Y and Z directions.

In Fig. 9a represents the surface of the non-textured cladding layer, with the main residual stress values ranging from 237.33 to 355.99 MPa. (b) Represents the surface of the pre-set textured cladding layer, with the main residual stress values ranging from 226.41 to 389.72 MPa. (c) Represents the bottom surface of the non-textured cladding layer, with the main residual stress values ranging from 355.99 to 533.98 MPa. (d) Represents the bottom surface of the pre-set textured cladding layer, with the main residual stress values ranging from 226.41 to 389.72 MPa. From Fig. 8, it can be observed clearly that the residual stress on the bottom surface of the pre-set textured cladding layer is relatively small. However, the difference in residual stress between the surface of the cladding layer and the surface of the non-textured cladding layer is relatively small, and in some cases, it is even higher than that of the non-textured cladding layer surface. Therefore, to explore the distribution of residual stress in the two types of cladding layers, the stresses in the X, Y, and Z directions are extracted separately, as shown in Fig. 10.

As shown in the figure above, the residual stress curves in the X direction for both textured and non-textured cladding layers exhibit a symmetrical relationship, and the two curves have similar overall trends. However, in the X direction, the residual stress in the pre-set textured cladding layer is consistently lower than that in the non-textured cladding layer. The average residual stress in the X direction for the non-textured cladding layer is 450.62 MPa, while the average residual stress in the X direction for the pre-set textured cladding layer is 293.62 MPa. The residual stress in the X direction for the pre-set textured cladding layer is reduced by approximately 34.84% compared to the non-textured cladding layer. The average residual stress in the Y direction for the textured and non-textured cladding layers is reduced by 3.94%. In the range of 0–0.6 mm, the residual stress in the Y direction for the pre-set textured cladding layer is greater than that for the non-textured cladding layer, but the difference between them is relatively small. In the range of 0.6–1.2 mm, the residual stress in the Y direction for the pre-set textured cladding layer is less than that for the non-textured cladding layer, which represents a reduction of about 53.51% at the bottom of the non-textured cladding layer. Residual stress curves in the Z direction generate significant residual stress at the beginning and end of the cladding process. The difference in residual stress between the textured and non-textured cladding layers is relatively small. This may be attributed to the fact that the prepared texture is a groove, and both the starting and ending positions during the melting process are at the edge of the workpiece. The droplets are affected by gravity and other forces, leading to the occurrence of significant residual stress. Apart from these two positions, the residual stress in the Z direction for the pre-set textured cladding layer is consistently lower than that for the non-textured cladding layer. Their respective average values are 509.96 MPa and 253.85 MPa. The pre-set textured cladding layer shows a reduction of approximately 50.22% in residual stress compared to the non-textured cladding layer. This further validates that the pre-set texture technique can effectively reduce residual stress in the cladding layer, improve the mechanical properties at the metallurgical interface of the cladding layer, and enhance the quality of the cladding layer.

### Analysis of fluid field results

**Figure 11:**
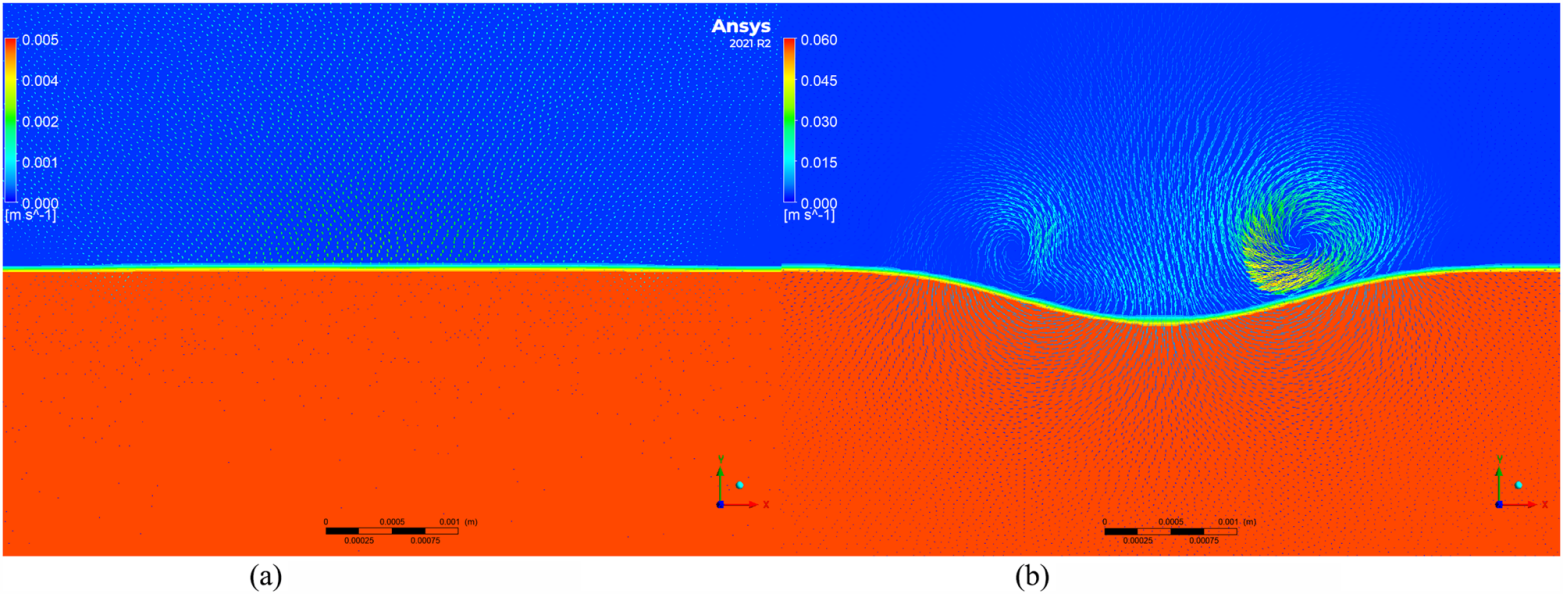
0.01 s with and without texture melt pool flow rate vector diagram. (**a**) Non surface texture. (**b**) Preset surface textures.

**Figure 12:**
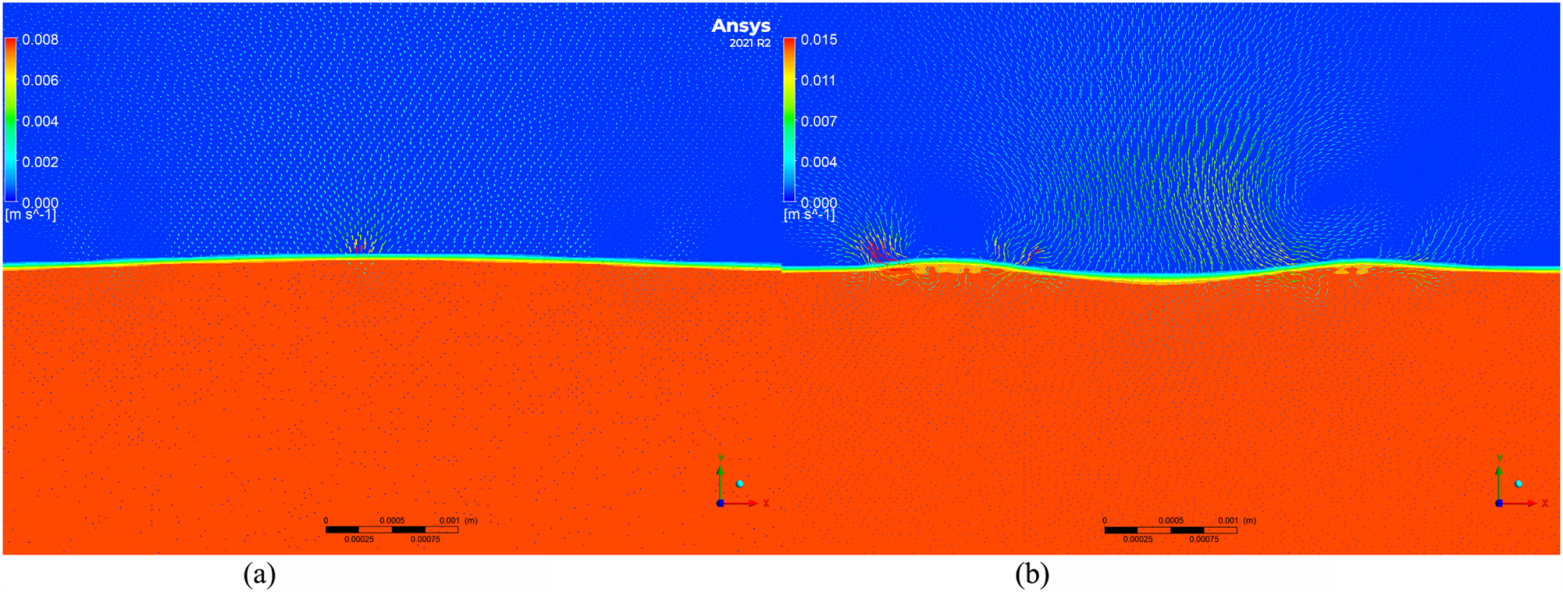
0.05s with and without texture melt pool flow rate vector diagram. (**a**) Non surface texture. (**b**) Preset surface textures.

**Figure 13:**
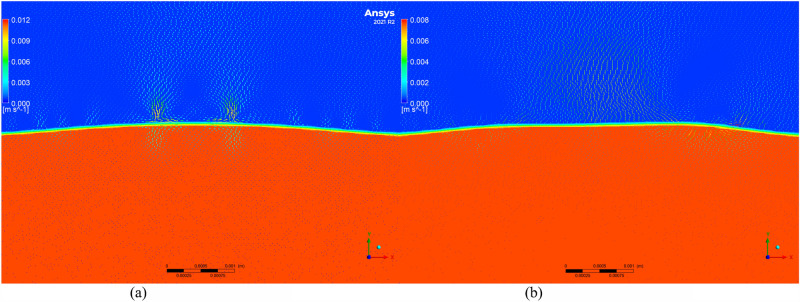
0.10s with and without texture melt pool flow rate vector diagram. (**a**) Non surface texture. (**b**) Preset surface textures.

**Figure 14:**
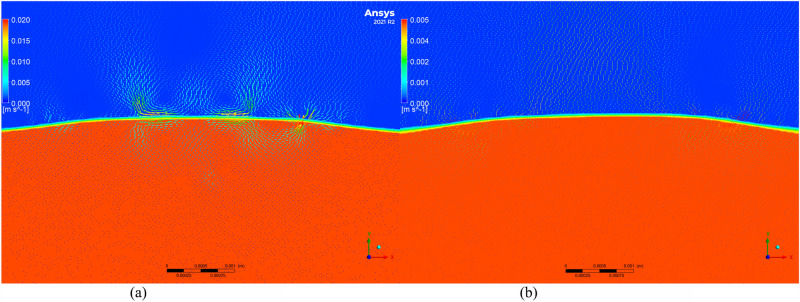
0.15 s with and without texture melt pool flow rate vector diagram. (**a**) Non surface texture. (**b**) Preset surface textures.

**Figure 15:**
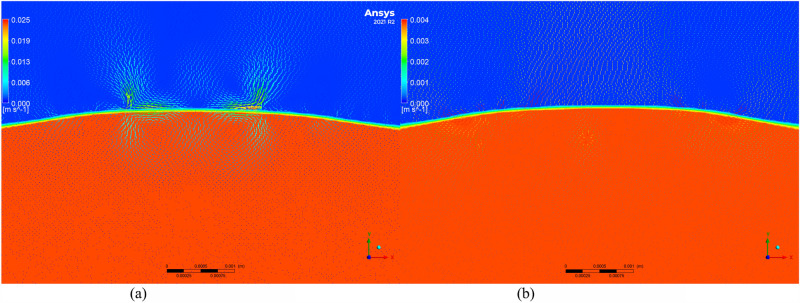
0.20 s with and without texture melt pool flow rate vector diagram. (**a**) Non surface texture. (**b**) Preset surface textures.

To further investigate the changes in residual stress and to understand the microscopic movement and equilibrium of the molten pool, the Volume of Fluid (VOF) model was employed to simulate the molten pool. Velocity vector maps of molten pools with and without texture were extracted, as shown in Figs. 11, 12, 13, 14 and 15. Blue represents air, and red represents the substrate. Each figure compares the velocity vectors between textured and non-textured molten pools.

At 0.01s, the non-textured molten pool exhibited convection at the air position with a maximum velocity of 0.005 m/s, while the internal flow velocity ranged from 0 to 0.002 m/s. The textured molten pool exhibited a dual-vortex effect at the air position with a maximum velocity of 0.06 m/s, and the internal flow velocity ranged from 0 to 0.03 m/s. Due to the good accessibility of the laser, the pre-texturing on the substrate surface effectively increased the contact area between the laser and the substrate. This resulted in a rapid temperature rise to the melting point on both sides of the texture, and there was a certain height difference between the sides and the bottom. Liquid metal on both sides slid down to the bottom, resulting in a significant flow velocity at the beginning of the melting. The difference in air position flow velocity is due to the presence of texture, which affects the surface morphology of the substrate. Air convection occurs at different positions. Non-textured melting only acts on the substrate surface, forming natural convection, while pre-textured melting acts on the bottom of the texture, in contact with the sides of the texture, thereby forming a dual-vortex effect.

At 0.05 s, the maximum flow velocity at the air position for the non-textured molten pool increased to 0.008 m/s, while the maximum flow velocity at the air position for the pre-textured molten pool decreased to 0.015 m/s, and the dual-vortex effect reduced. Observing the velocity vector map at this time, with the addition of powder, the textured morphology gradually disappeared. At this point, the maximum flow velocities at the air position for the two melting methods were relatively close. Inside the non-textured molten pool, the internal flow velocity ranged from 0 to 0.005 m/s, while inside the pre-textured molten pool, the internal flow velocity ranged from 0 to 0.011 m/s. Compared to the 0.01 s moment, the internal flow velocity in the non-textured molten pool gradually increased, while in the pre-textured molten pool, it gradually decreased.

At 0.10 s, the maximum flow velocity at the air position for the non-textured molten pool is 0.012 m/s, while for the pre-textured molten pool, the maximum flow velocity at the air position is 0.008 m/s, and the dual-vortex effect completely disappears. At this point, the textured morphology disappears completely, similar to the non-textured starting moment, and there is no dual-vortex effect at the air position. The internal flow velocity in the non-textured molten pool ranges from 0 to 0.01 m/s, while in the pre-textured molten pool, the internal flow velocity ranges from 0 to 0.006 m/s. Compared to the 0.05 s moment, the internal flow velocity in the non-textured molten pool gradually increases, while in the pre-textured molten pool, it gradually decreases, consistent with the above trend.

At 0.15 s, the maximum flow velocity at the air position for the non-textured molten pool is 0.02 m/s, while for the pre-textured molten pool, the maximum flow velocity at the air position is 0.005 m/s. At this point, the air velocity of the non-textured molten pool shows a decreasing trend compared to 0.10 s, while the air velocity of the pre-textured molten pool remains basically unchanged. The internal flow velocity in the non-textured molten pool ranges from 0 to 0.018 m/s, while in the pre-textured molten pool, the internal flow velocity ranges from 0 to 0.004 m/s. Compared to 0.10 s, the internal flow velocity in the non-textured molten pool continues to increase, while the pre-textured surface texture continues to decrease.

Compared to the 0.15s moment, the air and molten pool velocities at the air position and molten pool position in the non-textured overlay process remain basically unchanged at 0.20s. In summary, during the formation process of the non-textured molten pool, both air and molten pool velocities show an increasing trend and then basically remain unchanged. In contrast, the air and molten pool velocities in the pre-textured overlay process both show a decreasing trend and then basically remain unchanged. Through the analysis of internal flow velocity in the molten pool, it is observed that the internal flow velocity in the non-textured molten pool increases with time due to the inertia of the fluid. The increase in the flow velocity of the liquid metal will lead to the formation of a vortex effect inside the liquid metal, and this vortex effect will result in an uneven temperature distribution inside the molten pool, thereby producing a large temperature gradient, consistent with the numerical simulation results of the temperature field in the previous section. The presence of temperature gradients will cause convection movement inside the molten pool, intensifying the degree of fluid mixing and leading to a more significant temperature difference in the liquid at different locations. This, in turn, will result in the formation of larger residual stresses in the overlay. In contrast, the internal flow velocity in the pre-textured molten pool decreases with time, and the decrease in the flow velocity inside the molten pool leads to the absence of the vortex effect. The internal temperature distribution becomes more uniform, the temperature gradient decreases, and subsequently, the residual stresses in the overlay are smaller, which is consistent with the numerical simulation results in the previous section.

## Experiment

### Analysis of fluid field results

#### Preparation of overlay

**Figure 16:**
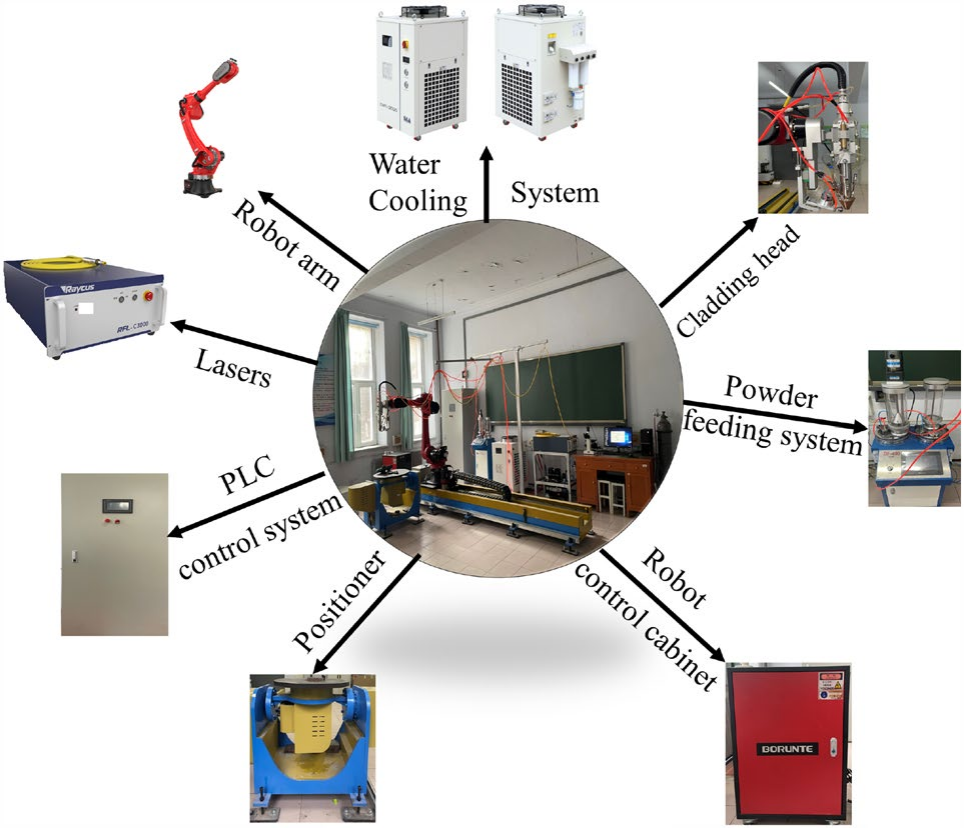
Laser cladding equipment diagram.

The experimental substrate is 45 steel, and the powder used is 316L stainless steel. An RFL-C3000S fiber laser with a wavelength of 1080 nm and a spot diameter of 4 mm is employed, featuring a rated output power of 3000 W, a power adjustment range of 10–100%, and a modulation frequency of 1–5000 Hz. The cladding experiment is performed using a synchronous powder feeding method, and the cladding equipment is depicted in Fig. 16. The protective gas used is high-purity argon. Before overlaying, the rust and oxide film on the substrate surface are removed, and the overlay powder is dried at 120 °C for 10 h. The process parameters used in this study are as follows: laser power of 600 W, scanning speed of 4 mm/s, defocusing distance of 20 mm, and overlay length of 30 mm.

#### Texture preparation

**Figure 17:**
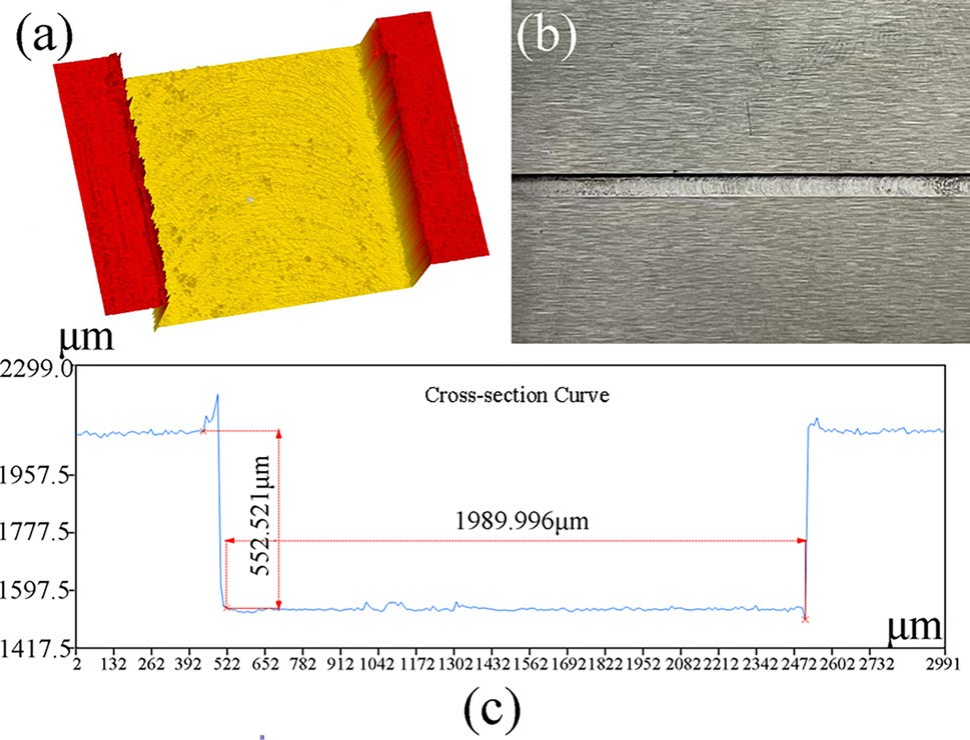
Surface morphology of the texture.

The grooved texture was processed using the BMDX-5040 Baoma engraving and milling machine. The processed texture morphology was measured using the KS-1000 3D digital microscope, as shown in Fig. 17, where (a) is the 3D model, (b) is the physical texture image, and (c) is the depth profile of the texture.

#### Laser melting and deposition process

**Figure 18:**
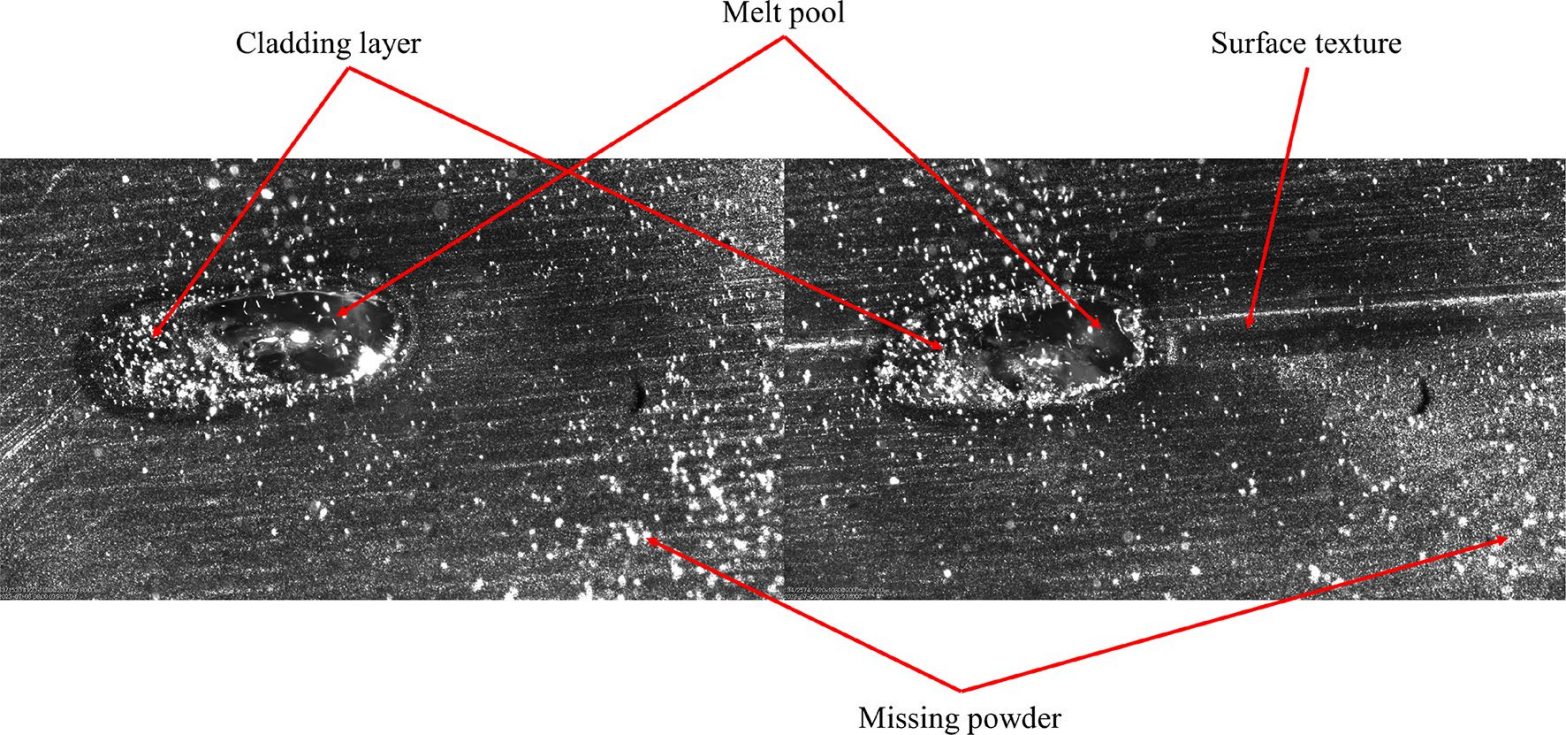
High-speed photography of laser melting and deposition.

Utilizing high-speed photography with appropriate frame rate and resolution allows capturing key processes and details of laser melting and deposition in both textured and non-textured states, as shown in Fig. 18.

Utilizing high-speed photography, we captured the process of metal powder melting. Upon observing Fig. 18, it can be noted that metal powder gradually melts under the irradiation of the laser beam, forming a molten pool, which quickly solidifies to create the deposited layer. The introduction of texture alters the heat conduction paths within the molten pool, providing a larger surface area and more contact interfaces. This increases the conduction path for heat between the molten pool and the substrate, leading to a more rapid and uniform diffusion of heat within the molten pool. Consequently, the molten pool exhibits a relatively flattened shape, whereas the molten pool in the non-textured laser melting deposition assumes a semi-ellipsoidal shape.

### Results and analysis

#### Residual stress results and analysis

**Figure 19:**
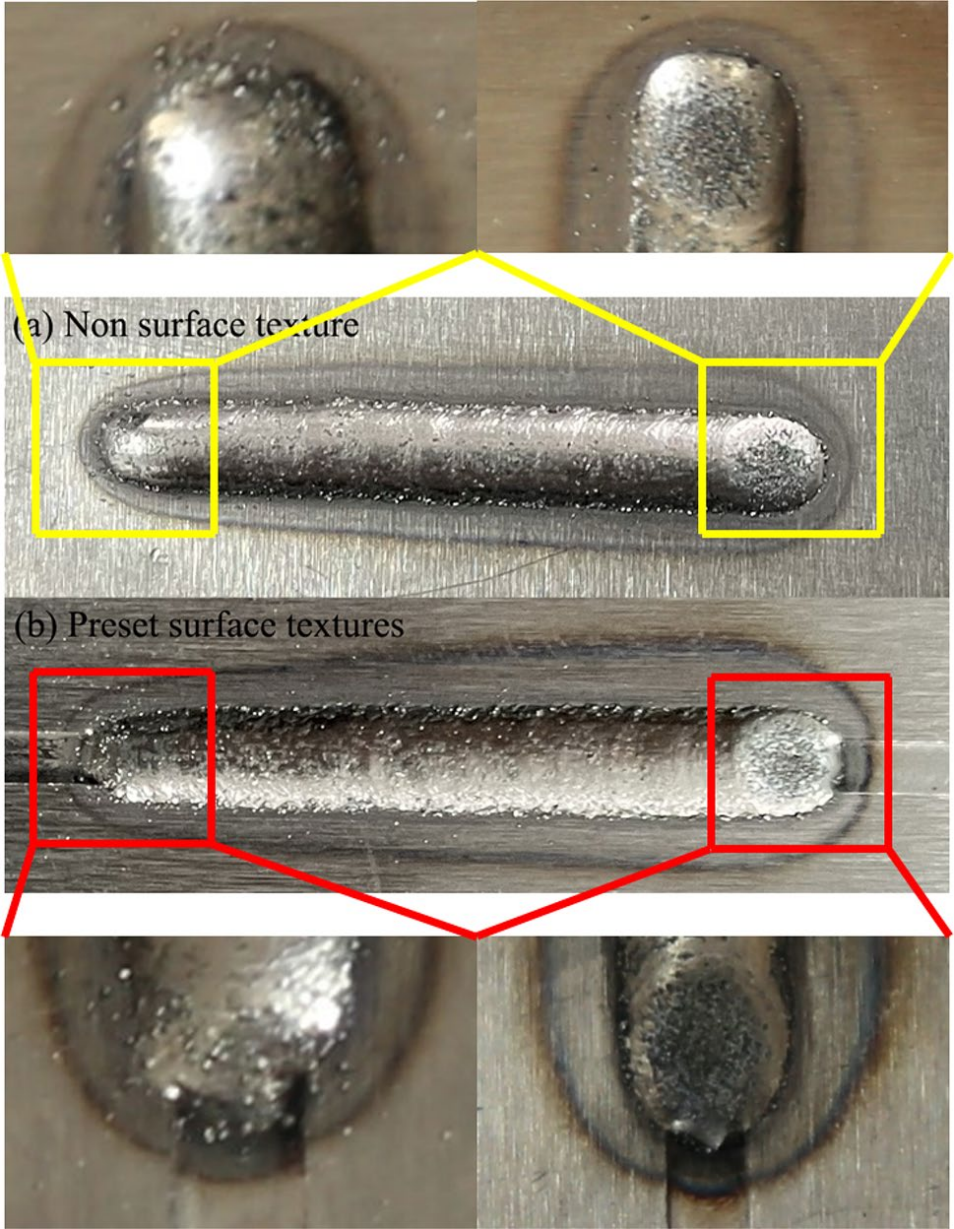
Physical appearance of the deposited layers with and without Texture.

The laser deposition experiments with and without texture were conducted using the optimal process parameters, resulting in the physical appearance of the deposited layers as shown in Fig. 19.

**Figure 20:**
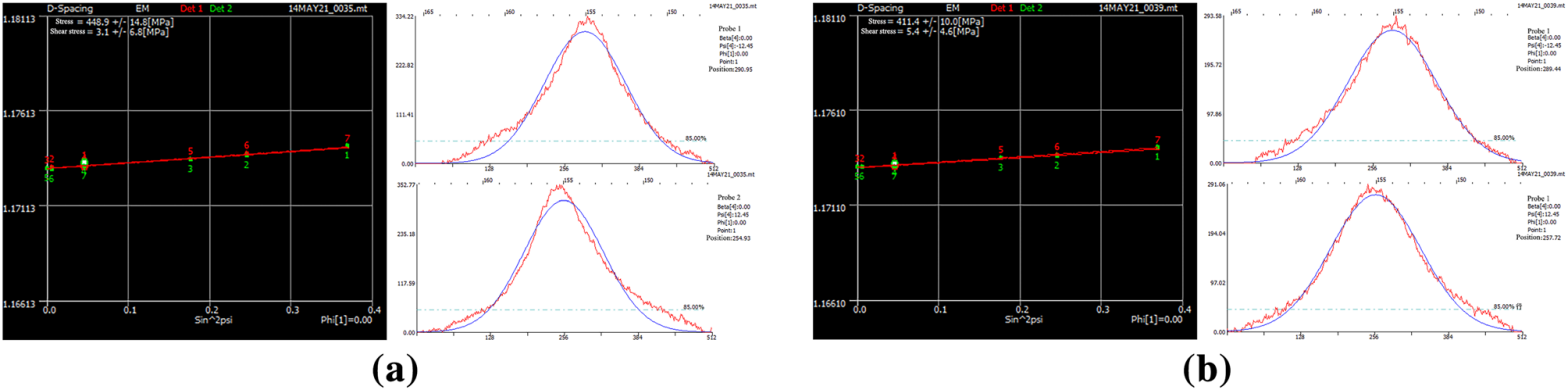
Residual stress measurement results at starting and ending positions. (**a**) Starting position. (**b**) Ending position.

Upon observing Fig. 19, it is evident that there is a phenomenon of droplet sliding at the starting and ending positions of the pre-textured deposited layer, while the non-textured deposited layer does not exhibit this phenomenon. Droplet sliding at both ends of the deposited layer can lead to surface irregularities, causing unevenness and stress concentration. In irregular shapes, variations in crystal orientation and grain size may occur, potentially leading to lattice defects and significant residual stresses, consistent with the numerical simulation results. Residual stress at the start and end positions of the predefined texture cladding layer was measured. The measurements were conducted using an X-ray residual stress device with a rated voltage of 25 kV, a Cr K-alpha X-ray tube, and a wavelength of 2.291. The measurement conditions included a rotation angle from 25° to − 25°, an exposure time of 1 s, 10 exposures, and a 1 mm aperture. The results are shown in Fig. 20.

Figure 20 shows that the residual stress at the start position of the predefined texture cladding layer is 448.9 MPa, and at the end position, it is 411.4 MPa. The numerical simulation indicates a residual stress of 420.16 MPa at the start position and 387.32 MPa at the end position, with errors of approximately 6.40% and 5.85%, respectively. The small discrepancies between the simulation and the experimental results further validate the accuracy of the numerical simulation.

**Figure 21:**
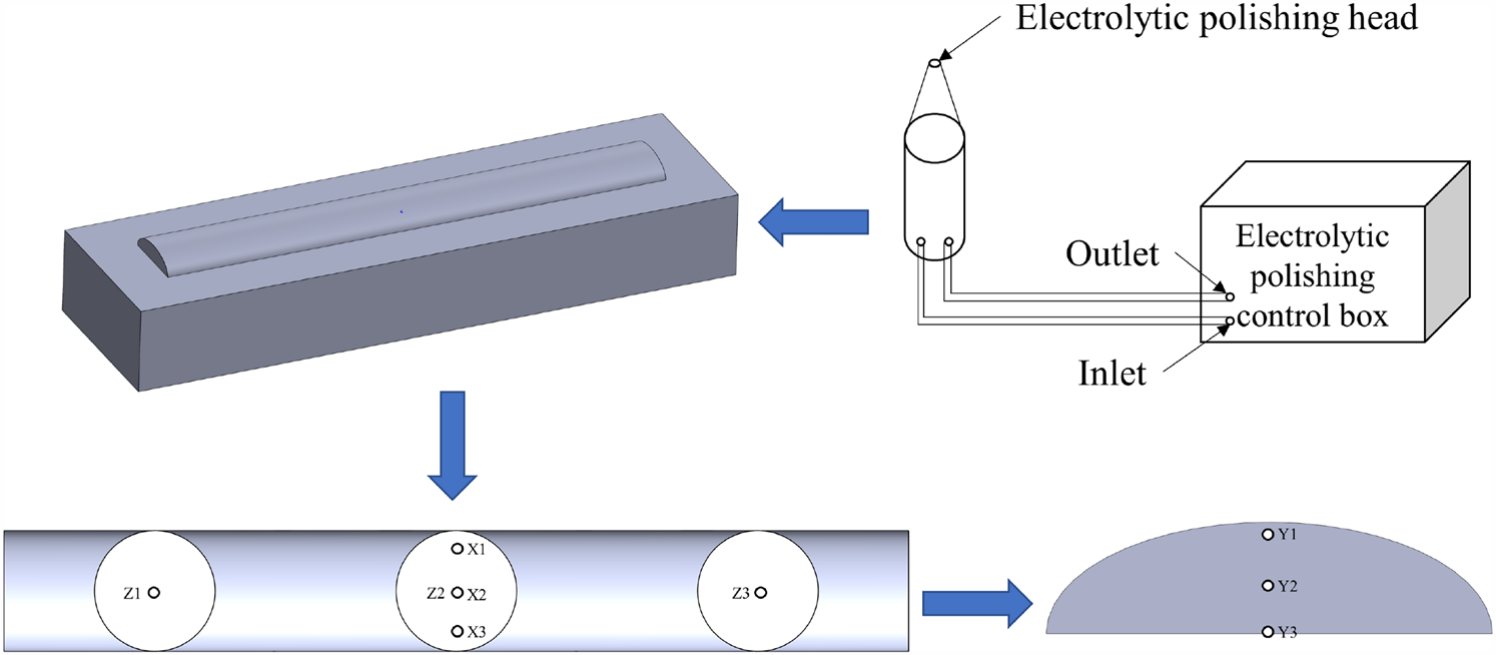
Electrolytic polishing sampling principle.

**Figure 22:**
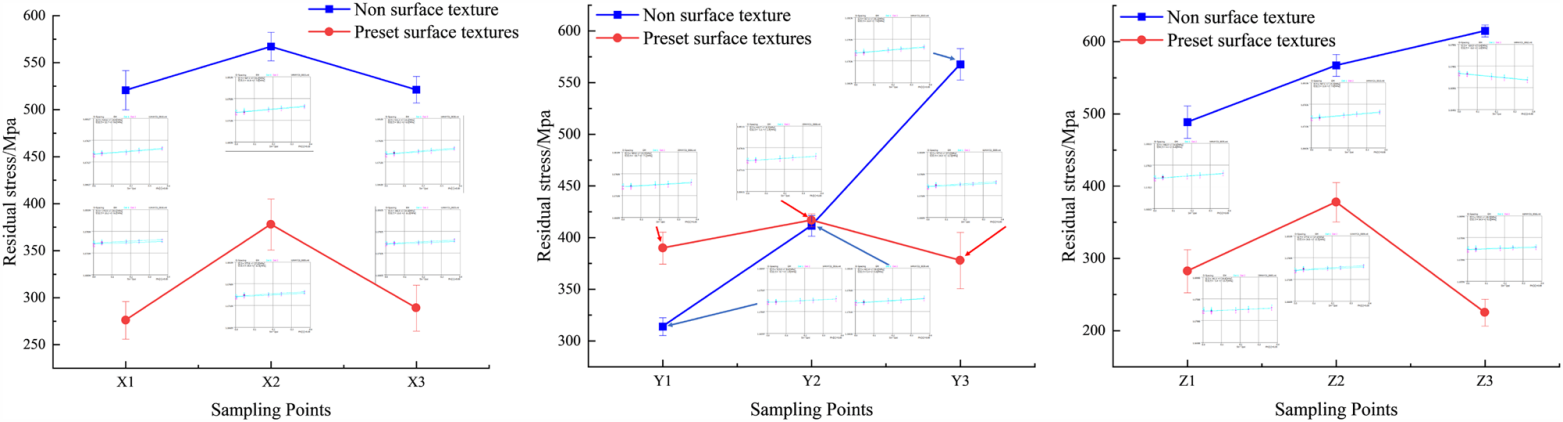
Residual Stress in X, Y, and Z directions with and without texture.

Excluding the starting and ending positions of the overlay layer, the residual stress in the remaining parts of the overlay layer was measured. Electrolytic polishing equipment was used to polish the textured and non-textured overlay layers. By controlling the polishing time and frequency, the sampling points for residual stress were controlled, enabling the measurement of residual stress in the X, Y, and Z directions of the overlay layer. The sampling method is illustrated in Fig. 21. The measurement results of residual stress at sampling points in the textured and non-textured overlay layers are shown in Fig. 22.

Residual stresses during laser cladding are primarily formed by the superposition of thermal stress, cooling stress, microstructure stress, and shape stress. However, uncertainties in material parameters, setting of surface and boundary conditions, and assumptions and simplifications in the model during numerical simulation can affect the results. The difficulty of fully incorporating temperature gradients, cooling rates, and solidification processes into the numerical simulation leads to slightly lower numerical results compared to actual measurements. Nonetheless, the actual processing results generally align with the trends in numerical simulation, and the numerical differences are relatively small.

In the non-textured cladding layer, the actual residual stresses in the X, Y, and Z directions are 536.30 MPa, 430.97 MPa, and 556.87 MPa, respectively. In the predefined texture cladding layer, the actual residual stresses in the X, Y, and Z directions are 314.16 MPa, 394.77 MPa, and 295.03 MPa, respectively. Compared to the non-textured cladding layer, the residual stresses in the predefined texture cladding layer decrease by approximately 41% and 47% in the X and Z directions, respectively, while the decrease in the Y direction is smaller, at approximately 8.4%.

This observation is consistent with the numerical simulation results of the thermal stress and fluid field simulation. Considering both actual measurements and the numerical simulation results, pre-existing texture demonstrates a significant advantage in reducing the residual stresses at the metallurgical bond between the clad layer and the substrate, especially in the X and Z directions.

#### Surface roughness analysis

**Figure 23:**
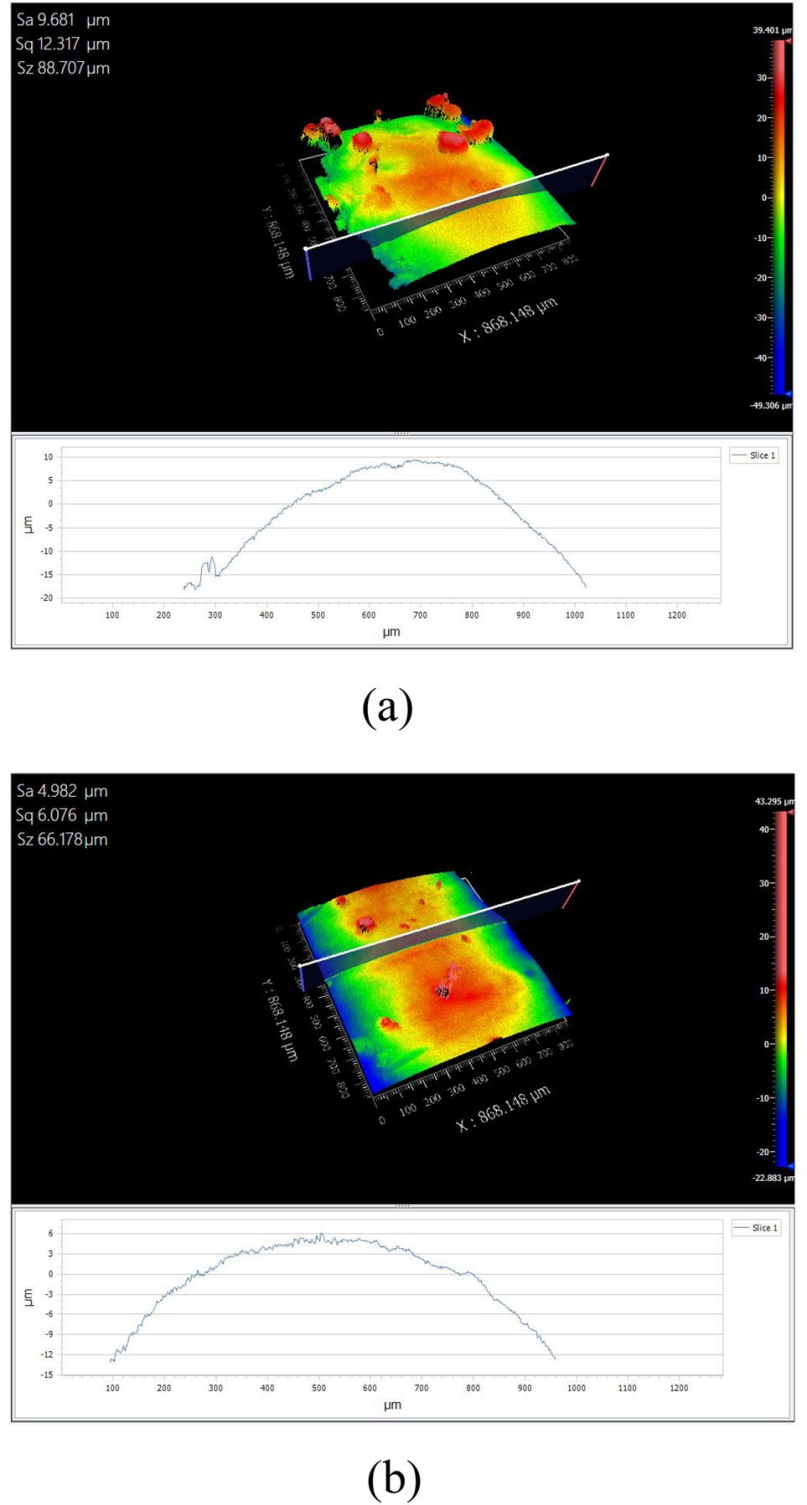
Surface roughness measurement results with and without texture. (**a**) Surface roughness of texture-free clad layer. (**b**) Surface roughness of pre-textured clad layer.

The surface roughness of the clad layer refers to the unevenness or roughness of the clad layer's surface. It can be described by various parameters. In this study, the ZYGO three-dimensional contour scanner was used to measure Sa, Sq, and Sz values of the clad layers with and without texture, aiming to assess the surface roughness. The measurement results are presented in Fig. 23.

From Fig. 23, it can be observed that the Sa value of the non-textured cladding layer is 9.681 μm, Sq value is 12.317 μm, and Sz value is 88.707 μm, while the Sa value of the pre-textured cladding layer is 4.982 μm, the Sq value is 6.076 μm, and the Sz value is 66.178 μm. Observing these data, it is evident that the pre-textured cladding layer exhibits lower roughness parameters (Sa, Sq, Sz) compared to the non-textured cladding layer, indicating that pre-texturing can effectively improve the surface quality of the cladding layer and reduce the surface roughness. There is a certain interaction between surface roughness and residual stress. Residual stress can cause bending, deformation, or shrinkage of the material, thereby altering the shape and undulation of the surface. If significant residual stress is present, it may result in plastic deformation, causing depressions, wrinkles, and increased surface roughness, and even lead to crack initiation, ultimately causing failure. Cladding layers with smaller surface roughness are typically more uniform and smoother. A smoother surface helps to distribute stress uniformly, reducing stress concentration and surface defects.

#### Microstructure analysis

**Figure 24:**
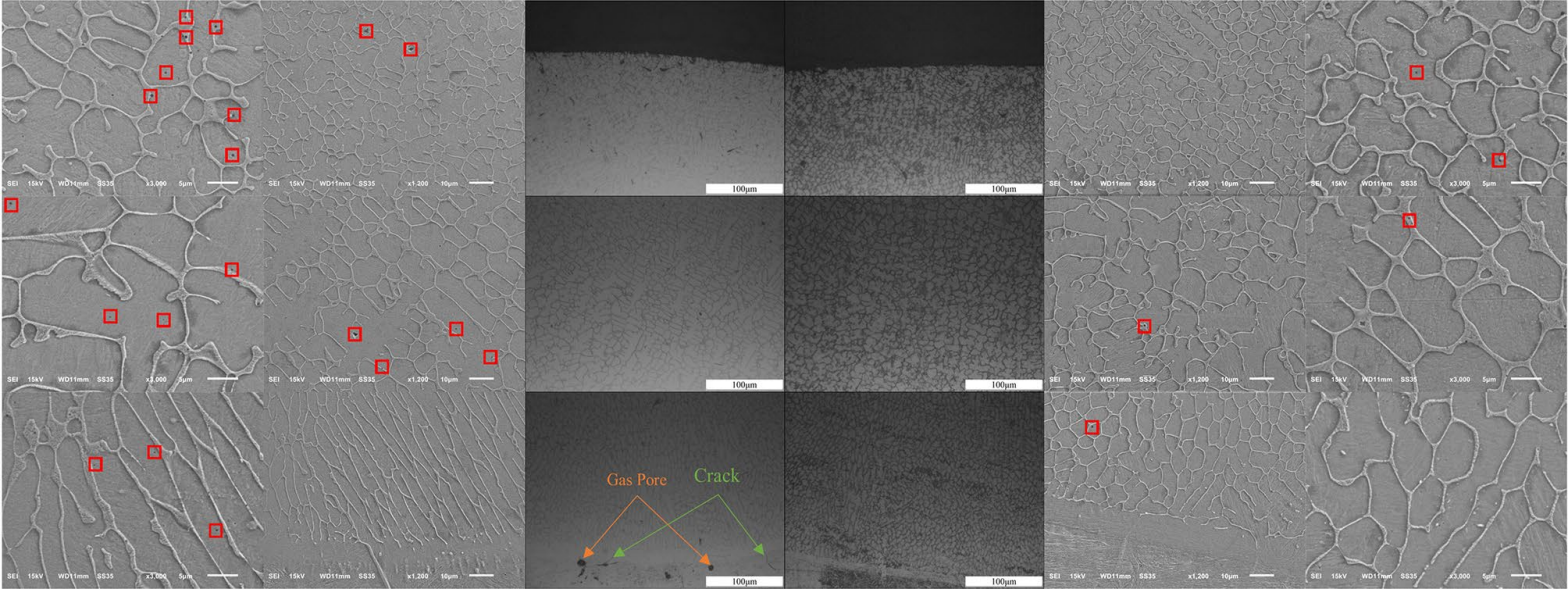
Microstructure of clad layers with and without texture. The red outline highlights the pores.

To delve deeper into the internal structure of the clad layer with and without texture, the cross-sections of the clad layer were corroded for 3 s using quiescent aqua regia after a 20-min immersion. The samples were then observed under a metallographic microscope to examine the metallographic structure, as depicted in Fig. 24.

The left side of Fig. 24 shows metallographic images at different magnifications of the top, middle, and bottom of the non-textured cladding layer, while the right side shows images of the predefined textured cladding layer. Figure 24 and Table 4 indicate that the grain sizes at the top, middle, and bottom of the predefined textured cladding layer are smaller than those of the non-textured cladding layer. The top is the part of the cladding layer that first contacts the substrate, resulting in a higher cooling rate. This higher cooling rate limits the grain growth rate, promoting the formation of smaller grains. Additionally, the predefined texture provides more nucleation sites, increasing the number of nuclei and restricting grain growth. Thus, the grain size at the top of the predefined textured cladding layer is smaller than that at the top of the non-textured cladding layer. Compared to the top and bottom, the cooling rate in the middle is slightly lower. However, the predefined texture increases the contact area between the middle of the cladding layer and the substrate, accelerating the cooling rate and resulting in smaller grain sizes. The bottom is the position where metallurgical bonding with the substrate occurs, resulting in significant heat conduction. When grooves are present at the bottom, they melt first, forming a larger contact area with the substrate and increasing the cooling rate. This larger contact area provides more solidification nuclei and nucleation sites, promoting the formation of cellular grains and inhibiting the growth of equiaxed grains, thereby improving the performance of the metallurgical bonding zone in laser cladding. The non-textured cladding layer exhibits numerous pores in both the top and middle regions, with pores and cracks at the bottom. In contrast, the predefined textured cladding layer has fewer pores in the top and middle regions and no significant defects at the bottom. The lower cooling rate at the bottom of the non-textured cladding layer prevents gas in the molten pool from escaping smoothly, leading to pore formation. The higher cooling rate at the bottom of the predefined textured cladding layer facilitates rapid solidification, reducing the formation of pores and cracks. Additionally, the predefined groove texture on the substrate surface enhances the metallurgical bonding strength between the substrate and the cladding layer, effectively preventing crack propagation and reducing pore formation.

**Table 4:** Grain sizes in different regions of the laser cladding layer, with and without predefined texture.

Specimen	Texture-free	Mean value	Predefined texture	Mean value
Upper part (μm^2^)	52.4489	68.4557	69.9795	56.9659
	82.5714		48.8163	
	70.3469		52.1020	
Central part (μm^2^)	317.0408	248.5973	102.4490	103.6122
	150.0612		123.3061	
	278.6900		85.0816	
Lower part (μm^2^)	78.6989	103.5714	51.2755	68.7500
	93.2398		90.5612	
	138.7755		64.4132	
Mean value (μm^2^)	140.2081		76.4427	

#### Hardness analysis

**Figure 25:**
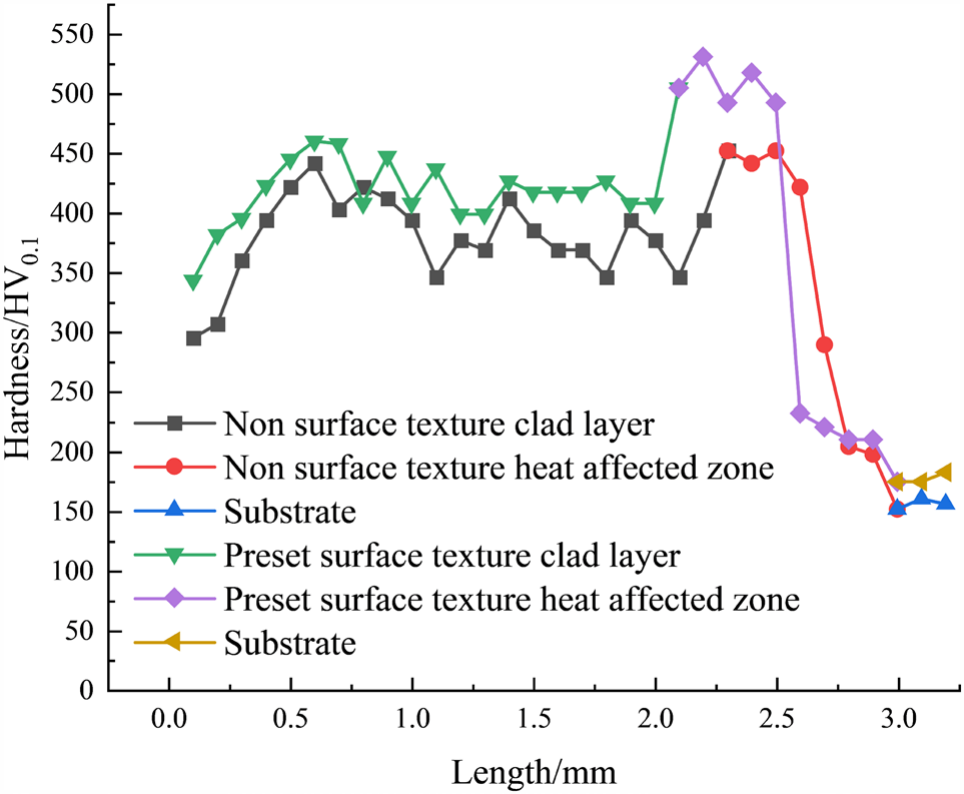
Hardness variation curves with and without texture.

Vickers hardness tester was used to measure the hardness of the texture-free clad layer, and the results are shown in Fig. 25. At the top position of the clad layer (0.1–0.4 mm), the hardness of the texture-free clad layer gradually increased, from 343.5 HV_0.1_ to 423.1 HV_0.1_ and from 295.1 HV_0.1_ to 394.1 HV_0.1_, but the hardness of the texture-prepared clad layer was higher than that of the texture-free clad layer. In the middle position of the clad layer (0.5–1.6 mm), the hardness of the texture-prepared clad layer was relatively stable, fluctuating in the range of 460.3 HV_0.1_ to 417.4 HV_0.1_, while the hardness of the texture-free clad layer varied in the range of 441.8 HV_0.1_ to 369 HV_0.1_, with larger fluctuations and lower than the hardness of the texture-prepared clad layer. The hardness variation at the bottom position (1.7–2 mm) of the texture-prepared clad layer is small, maintaining stability in the range of 417.4 HV_0.1_ to 408.1 HV_0.1_, while the hardness at the bottom position (1.7–2.1 mm) of the texture-free clad layer fluctuates in the range of 394.1 HV_0.1_ to 346.3 HV_0.1_. In the heat-affected zone, the maximum hardness of the texture-free clad layer and the texture-prepared clad layer is 531.3 HV_0.1_ and 452.4 HV_0.1_, respectively. Near the cladding layer, the hardness is generally higher, while near the substrate, the hardness is lower and closer to that of the substrate. This is due to the presence of a transition zone between the cladding layer and the substrate, where the composition and microstructure are a mixture of the cladding layer and the substrate material, exhibiting a gradient from the high hardness of the cladding layer to the lower hardness of the substrate. In summary, the analysis of residual stress and microstructure indicates that the predefined texture improves the microstructure and surface characteristics of the cladding layer through the introduction of fine grains and residual stress, thereby enhancing the hardness performance of the material.

### Analysis of the mechanism of pre-set texture

**Figure 26:**
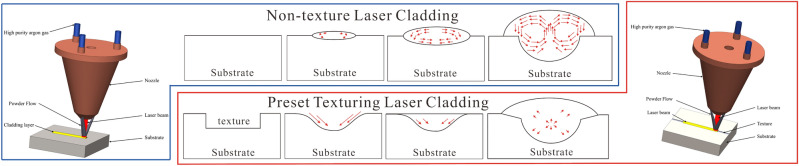
Mechanism of pre-set texture effect.

Pre-set texture is a subtractive manufacturing process that, by etching, cutting, or processing specific structures and patterns on the substrate surface, can alter material characteristics such as thermal conduction paths, fluid flow, and mechanical response. This leads to improved surface quality, enhanced wettability, and reduced friction resistance. The combination of pre-set texture and laser cladding achieves a process of additive and subtractive manufacturing. The introduction of texture alters the heat conduction path between the melt pool and substrate, achieving more uniform heat distribution and reducing temperature gradients. The texture structure provides additional heat dissipation area and heat transfer channels, enhancing heat diffusion and dissipation, resulting in a smoother temperature change within the melt pool and reduced temperature fluctuations. The texture structure also alters the flow path and velocity distribution of liquid metal, reducing velocity gradients and turbulence intensity. As shown in Fig. 26 (red arrows represent velocity vectors, with larger arrows indicating higher velocities), texture can eliminate the double vortex effect during the cladding process, leading to lower internal melt pool velocities and reducing the formation of structural non-uniformities and defects.

In conclusion, the combined additive and subtractive manufacturing process of pre-set texture with laser cladding allows for the creation of specific structures and morphologies on the substrate, by modifying temperature gradients, flow field distribution, and stress distribution during the cladding process. This enables control and improvement of the quality of the cladding layer. This composite manufacturing process leverages the advantages of both additive and subtractive techniques and offers a new approach to achieve high-quality and high-performance cladding layers.

## Conclusions

In this study, the combination of surface texturing technology with laser cladding technology resulted in the formation of a high-performance cladding layer. The conclusions are as follows:

1. Through multi-field coupled numerical simulations, it was found that pre-set texture effectively reduces temperature gradients during the cladding process, thereby creating a smoother thermal cycle curve. Pre-set texture on the substrate surface increased the unstable alternating thermal stresses during the cladding process, leading to a respective reduction of 34.84%, 3.94%, and 50.22% in residual stresses in the X, Y, and Z directions. Additionally, the internal flow velocity of the molten pool gradually decreased, preventing the formation of double-vortex flow effects, and enhancing the surface quality of the cladding layer.
2. Experimental observations of laser cladding with and without texture revealed droplet sliding at both ends of the pre-set texture cladding layer. This phenomenon resulted in higher residual stresses at the start and end positions of the cladding layer, consistent with the numerical simulation results of the stress field. However, at other positions, the pre-set texture cladding layer exhibited reductions of 41.42%, 8.04%, and 47.02% in residual stresses in the X, Y, and Z directions, respectively, compared to the non-textured cladding layer.
3. The Sa, Sq, and Sz values of the non-textured cladding layer are 9.681 μm, 12.317 μm, and 88.707 μm, respectively, while those of the pre-set texture cladding layer are 4.982 μm, 6.076 μm, and 66.178 μm. The roughness values (Sa, Sq, Sz) of the pre-set texture cladding layer are all smaller than those of the non-textured cladding layer, indicating that pre-set texture effectively improves the surface quality of the cladding layer and reduces surface roughness. The introduction of texture technology effectively increases the cooling rate during the cladding process, resulting in smaller grain sizes at the top, middle, and bottom of the pre-set texture cladding layer compared to the non-textured cladding layer. No significant defects are observed at the metallurgical bonding positions, indicating that the pre-set texture aids in controlling grain size and morphology, increase the hardness of the cladding layer, and consequently improve the quality of the cladding layer.

## Data Availability

All data generated or analyzed during this study are included in this published article.
